# Seed Treatment With Jasmonic Acid and Methyl Jasmonate Induces Resistance to Insects but Reduces Plant Growth and Yield in Rice, *Oryza sativa*

**DOI:** 10.3389/fpls.2021.691768

**Published:** 2021-08-16

**Authors:** Santhi Bhavanam, Michael Stout

**Affiliations:** Department of Entomology, Louisiana State University Agricultural Center, Baton Rouge, LA, United States

**Keywords:** plant elicitors, jasmonates, induced resistance, trade-offs, rice water weevil

## Abstract

When applied exogenously to plants, jasmonates [i.e., jasmonic acid (JA) and methyl jasmonate (MeJA)] increase plant resistance against herbivores, and their use in pest management has been suggested. For integration into pest management programs, the benefits of the resistance induced by jasmonates must outweigh the costs of jasmonates on plant growth and yield. A previous field study in rice found that seed treatment with MeJA reduced densities of the rice water weevil, *Lissorhoptrus oryzophilus,* but also reduced plant growth. Yields from MeJA plots were similar to yields from control plots. Because this study was conducted under field conditions with natural levels of pest populations, it was unclear whether effects on growth and yield were due to direct effects of MeJA treatment on the plant or due to lower reductions in rice water weevil densities. Therefore, the present study was designed to characterize the effects of JA and MeJA seed treatment on rice plant growth and yield in a pest-free environment under greenhouse conditions. Seed treatment with 2.5 mM JA and 2.5 mM MeJA enhanced resistance in rice plants to rice water weevils when plants were exposed to weevils 30 days after planting. Seed treatment with MeJA reduced seedling emergence and plant height at 4 and 14 days after planting, respectively, compared to JA and control treatments. However, numbers of tillers per plant at 45 days after planting and days to heading were unaffected by jasmonate seed treatment. Of four yield components (panicles per plant, filled grains per panicle, percent unfilled grains, and filled grain mass) that were measured, only filled grain mass was reduced by seed treatment. Plants grown from MeJA-treated seeds showed 31% lower grain masses compared to plants grown from control-treated seeds. Thus, the effects of seed treatment with MeJA on plant growth were stronger immediately post-treatment and subsided over time, such that plant growth mostly recovered 6 weeks after treatment. At maturity, MeJA may reduce one but not all components of yield. Despite similar effects on rice water weevil resistance, the negative effects of JA seed treatment on plant growth and yield were smaller compared to MeJA seed treatment.

## Introduction

Jasmonic acid (JA), its methylated derivative methyl jasmonate (MeJA) and its conjugate with isoleucine (JA-Ile), collectively referred to as jasmonates, are phytohormones that regulate several physiological and developmental processes in plants (Ali and Baek, 2020). Jasmonates play a critical role in plant resistance against insect pests, pathogens, and abiotic stresses (Ali and Baek, 2020). Feeding by chewing insects, necrotrophic pathogens, and certain types of abiotic stresses activate the JA signaling pathway (Raza et al., 2020), which stimulates direct defense in plants through formation of morphological structures, such as trichomes, or biochemical responses, such as production of plant secondary metabolites and resistance-related enzymes, all of which can interfere with preference and performance of insect pests (Walling, 2000). Moreover, jasmonate-induced defenses include changes in the qualitative and quantitative composition of plant volatile compounds that can directly affect herbivores and attract insect natural enemies; the latter can lead to increased parasitization and predation rates of herbivores, thereby providing indirect resistance to plants against herbivores (Lou et al., 2005; Okada et al., 2015).

Over the years, increased understanding of defense signaling pathways and induced resistance in plants has led to the development of natural or synthetic elicitors that can mimic responses to natural herbivory (Karban and Kuc, 1999). Exogenous application of JA, like feeding by chewing insects, rapidly increases the levels of endogenous JA, which in turn triggers the expression of defense-related genes (Pauwels et al., 2009; Benevenuto et al., 2019). Exogenous MeJA also elicits JA-related responses after MeJA is demethylated to JA in plants (Wu et al., 2008). Exogenous application of JA and MeJA through foliar sprays or soil drenching enhances resistance against a broad spectrum of insects in a wide range of agricultural crops under greenhouse and field conditions (Rodriguez-Saona et al., 2001; Choh et al., 2004; Hamm et al., 2010; Nabity et al., 2013; Gordy et al., 2015; Haas et al., 2018; Nouri-Ganbalani et al., 2018). Recently, studies have attempted to use application of JA or MeJA to seeds before germination to induce resistance in plants. Seed treatments with plant elicitors have mostly been investigated in tomato, *Solanum lycopersicum* L., with a few additional studies on cabbage, *Brassica oleracea* and rice, *Oryza sativa* L. These studies provide evidence that seed treatment with JA or MeJA can increase resistance against herbivores in plants (Worrall et al., 2012; Paudel et al., 2014; Strapasson et al., 2014; Haas et al., 2018; Kraus and Stout, 2019). However, variations in JA-induced responses existed among cultivars within a plant species as well as among plant species as seeds of different varieties were not equally receptive to seed treatment with plant elicitors (Smart et al., 2013; Mouden et al., 2020). Moreover, unlike applications of plant elicitors by foliar spray or soil drench, which are usually made at or near the time of pest infestation, seed treatments are made before seeds are planted, and hence, the duration of induction of defenses by seed treatments is a critical consideration for the efficacy of seed treatments (Worrall et al., 2012; Haas et al., 2018; Kraus and Stout, 2019).

The utility of JA and MeJA as elicitors in insect pest management programs is potentially limited because, while exogenous jasmonates may induce resistance to pests, they may incur a cost to the plant by reducing plant growth and yield. These costs may occur through trade-offs in resource allocation; that is, plants have limited resources that can be invested either in growth or defense and increased allocation of resources to one trait limits the resources available for the other (Jimenez-Aleman et al., 2017; Guo et al., 2018). Thus, diversion of resources away from growth and to defense may have an impact on plant vegetative and reproductive growth. Another possible mechanism for reduced growth and yields in jasmonate-treated plants involves hormonal cross talk between JA signaling and other plant hormones (Hou et al., 2013). The costs of induced resistance to plants may be manifested in the form of delays in seed germination and post-germination seedling development, as seen in many cereal crops (Zwar and Hooley, 1986; Norastehnia et al., 2007; Lahuta et al., 2018) but not in cowpea, *Vigna unguiculata* (L.) Walp and soybean, *Glycine max* (L.) Merr (Muttucumaru et al., 2013). In wild mustard, *Brassica kaber* D.C., exogenous application of JA increased the activities of trypsin inhibitor (TI) and peroxidase (POD) and the levels of glucosinolates but also increased the time to first flower (Cipollini and Sipe, 2001). However, JA and MeJA application may not always incur costs to growth because of increased allocation to defense (Van Dam et al., 2004). A few studies have shown that, after initial reductions in plant growth and development, applications of jasmonates have no effect (Worrall et al., 2012) or a positive effect (Barbosa et al., 2008; Feng et al., 2012) on plant growth and yield. To integrate the use of plant elicitors in pest management programs, further studies are needed to characterize the effects of JA and MeJA application on plant growth and yield parameters under pest-free conditions, which may facilitate the development of strategies to mitigate negative effects of plant elicitors on plant growth and reproduction.

The rice water weevil, *Lissorhoptrus oryzophilus* Kuschel, is the most important insect pest of rice in the United States (Villegas et al., 2021). Adults feed on leaves of young rice plants leaving longitudinal scars on the leaf blades (Stout et al., 2002; Zou et al., 2004). Adult oviposition largely commences after flooding of rice fields (Stout et al., 2002). Eggs are laid inside the tissues of submerged leaf sheaths (Grigarick and Beards, 1965; Bowling, 1972). Upon egg hatching, first instars mine the tissue of leaf sheaths for a period of time before moving to the root system and feed on roots to complete larval and pupal development (Bowling, 1972). Pruning of roots by larvae negatively affects plant height, numbers of tillers and panicles, and ultimately, rice yields (Zou et al., 2004). The yield losses caused by rice water weevil infestation can reach up to 30% in untreated plots in southern United States (Villegas et al., 2021). Among different management practices, management of rice water weevil by chemical insecticides is the most commonly used tactic by growers (Aghaee and Godfrey, 2014), probably because of lack of effective alternative management tactics (Vyavhare et al., 2016). The use of plant elicitors to enhance resistance in plants to rice water weevil may reduce pest damage early in the season and thereby reduce the frequent application of insecticides, delay development of insect resistance to insecticides, and reduce harmful effects on non-target organisms.

In a previous study, Kraus and Stout (2019) showed that, under field conditions, MeJA-treated rice seeds enhanced resistance to the rice water weevil in rice plants, leading to reductions in population densities of rice water weevils on rice roots. The resistance induced by the MeJA seed treatment came, however, with a cost to plant growth. Seedling emergence was delayed, plant biomass was reduced, and time from emergence to heading increased in plants treated with MeJA plots relative to control plots. However, yields, measured in terms of per-panicle grain mass, were similar in plants grown from MeJA-treated seeds and plants from untreated seeds. In addition, yields in MeJA-treated plants were lower than yields from plants treated with insecticide to protect them from rice water weevils. These yield results were observed despite the fact that plants grown from MeJA-treated seeds harbored fewer numbers of rice water weevil relative to controls, raising the question of whether yields were directly impacted by MeJA treatment. The experimental design of the study was not sufficient to tease apart whether reductions in yield in MeJA-treated plants were due to elicitor treatment or lower reductions in RWW densities in the MeJA treatment compared to the insecticide treatment. Therefore, the present study was designed to characterize the effects of JA and MeJA seed treatment on plant vegetative and reproductive traits in pest-free conditions in a greenhouse, while simultaneously confirming the JA- and MeJA-induced resistance against rice water weevils.

## Materials and Methods

### Seed Treatment

Seeds of the rice cultivar “Cheniere,” obtained from the Louisiana State University (LSU) Agricultural Center Rice Research Station, Crowley, Louisiana, were used in all experiments. For seed treatments, 2.5 mM solutions of both JA (J0004-2200N-KF, Sigma-Aldrich, St. Louis, Missouri) and MeJA (Lot# MKCF2439, Sigma-Aldrich, St. Louis, Missouri) were prepared. The concentration of jasmonates used in this study was based on the results of Kraus and Stout (2019). Solutions were prepared by dispersing 75 μl of JA or 82 μl MeJA in 150 ml distilled water that contained 0.1% v/v (150 μl) of Tween-20 (Sigma-Aldrich, St. Louis, Missouri) in a 250 ml Erlenmeyer flask. The control solution included 0.1% v/v Tween-20 in the same volume of distilled water. The solutions in the flasks were mixed thoroughly on a magnetic stirrer before adding 50 g of rice seeds to the solutions. The flasks containing the seeds and treatment solutions were covered with aluminum foil, then placed on an orbital shaker, and gently shaken for 24 h at room temperature. Following the soaking, rice seeds were washed three times in distilled water, dried with paper towels, and used immediately in experiments.

### Greenhouse Experiment

A greenhouse experiment was conducted to characterize the effects of seed treatment on plant growth and yield while also verifying the induction of resistance to rice water weevil by seed treatments. The experiment was conducted in a greenhouse facility located near the campus of LSU, Baton Rouge, Louisiana. Plastic pots (1.9 L) that were filled with soil mix consisting of two parts topsoil, one part sand, and one part peat moss were used as the growth medium. Fifteen pots were prepared for each treatment (control, 2.5 mM JA, and 2.5 mM MeJA seed treatments) and, in each pot, five seeds were placed in the soil mix. Each pot was considered an experimental unit. Out of 15 pots prepared for each treatment, five pots were used to verify induction of resistance by the seed treatments and the remaining pots were used to characterize the effects of seed treatments on rice growth and yield. On the 14th day after planting, plants were thinned to one per pot and fertilized with approximately 2 g of 13-13-13 controlled release fertilizer (Carl Pool Products, Gladewater, Texas). Greenhouse temperature was maintained between 25 and 30°C. All plants were grown under ambient light and watered as needed.

Five pots of each treatment were used to verify the induction of resistance to rice water weevils by seed treatments as previously reported by Kraus and Stout (2019). This experiment was initiated 30 days after seed treatment. By this time, plants possessed one tiller. Rice water weevil adults were collected from research plots at LSU AgCenter H. Rouse Caffey Rice Research Station, Crowley, LA, on the day of experiment. Adults were kept in a Mason jar and provided with rice leaves and water. Five cylindrical cages, each consisting of a frame made of chicken wire (46 cm diameter × 61 cm height) covered with fine mesh, were placed on a wooden basin that was lined with a plastic pond liner to allow flooding of basins. A single pot from each of the three treatments was randomly selected and placed inside each cage; thus, each cage contained one pot of each of the three treatments. The wooden basin was filled with water up to a height of 25 cm. Nine adult rice water weevils (6 females and 3 males) were introduced into each cage, and the cage was covered with mesh. A total of five cages (replicates) were established in this manner. Adults were allowed to feed, mate, and oviposit inside the cages for 5 days. On day 5, the three pots inside a cage were removed and surviving adults found on the plants were killed. The plants were carefully removed from the soil, and roots were rinsed to remove the soil. Each plant was placed in a test tube that was filled with water and labelled appropriately. The test tubes with rice plants were brought to the lab and held in an environmentally-controlled room (25°C). The following day, each plant was vigorously shaken in the water in the test tube and the water was poured into a Petri dish. The numbers of first instar rice water weevils found in each dish were counted. Plants were returned to test tubes and tubes refilled with water. This procedure was repeated every day until no additional larvae were found in test tubes. The numbers of larvae emerging in each test tube were summed, and the sum was used for analyses.

To characterize the effects of seed treatment with JA and MeJA on rice plant growth and yield, several agronomically important plant traits were measured at appropriate time points during plant development from the 10 replicates of each treatment, as follows:

- Seedling emergence: All pots of each treatment were observed for seedling emergence (visible appearance of coleoptile above the soil) on the fourth day after planting. For each pot, the number of emerged seedlings was counted and percent emergence was calculated using the formula,
  $$\mathrm{Percent emergence}=\frac{\mathrm{Number of emerged seedlings}}{\mathrm{Number of seeds placed in}\;a\;\mathrm{pot}}\times 100$$
- Plant height: Plant height was measured on the 14th day after planting and before thinning. For all emerged seedlings in a pot, plant height from the top of the soil surface to the tip of the uppermost leaf was measured to the nearest 0.1 cm. Mean plant height per pot was calculated and used for analysis.
- Tillers: Data on numbers of tillers per plant were recorded on the 45th day after planting.
- Days to heading: Beginning on the 55th day after planting, plants were observed daily for heading (panicle exertion from the boot). For each plant, the date of first heading was noted and the number of days from planting to heading was calculated.
- Yield components: At harvest (removal of grain-bearing panicles from the plant), several components of plant yield were recorded. The number of panicles on each plant was counted. Then, all panicles on a plant were harvested and each panicle was placed in a separate labelled coin envelope. Panicles were oven-dried at 65°C for 7 days. After drying, individual panicles were hand-threshed. For each panicle, filled grains were separated from unfilled grains and the number of filled and unfilled grains was recorded. The same procedure was followed for all panicles of a plant. The number of filled grains and unfilled grains of a plant was totaled. Filled grains per panicle were calculated as below:
  $$\mathrm{Filled grains}\;\mathrm{per}\;\mathrm{panicle}=\frac{\mathrm{Total number of filled grains of a plant}\;}{\mathrm{Number of panicles of}\;a\;\mathrm{plant}}$$
  Percent unfilled grains were calculated as below:
  $$\mathrm{Percentage unfilled grains}=\frac{\begin{array}{l} \mathrm{Total numbers of unfilled}\\ \mathrm{grains of}\;a\;\mathrm{plant}\; \end{array}}{\begin{array}{l} \mathrm{Total numbers of grains}\;\\ \left(\mathrm{unfilled}+\mathrm{filled}\right)\mathrm{of}\;a\;\mathrm{plant} \end{array}}\times 100$$
  Mass of filled grains per plant was also measured.

### Laboratory Study

A separate study was conducted to assess in more detail the effects of JA and MeJA seed treatments on rice seed germination. Seeds were subjected to the three treatments described above. From each treatment, 20 seeds were randomly selected and placed in 4 rows of 5 seeds each in a square Petri dish (9 × 9 cm) that was lined with double-layered moistened germination paper (Anchor Paper Company, St. Paul, Minnesota). All seeds were oriented in the same direction. Seeds were covered with a VWR-light duty tissue wiper (VWR International LLC, Pennsylvania), and lids were placed on the Petri dishes. Nine Petri dishes for the control treatment (0 mM) and three Petri dishes for each of the other two treatments (2.5 mM MeJA and 2.5 mM JA) were prepared. Each Petri dish was treated as a replicate, and three replicates of each treatment were placed in separate plastic boxes (36 cm length × 20 cm width × 12.5 cm height). In addition, three Petri dishes of the control treatment were placed in each of the boxes with the dishes of the MeJA and JA treatments. Each plastic box was closed with a lid. To maintain conditions of high humidity, a wet paper towel was placed at the bottom of each plastic box before Petri dishes were placed on boxes. All the plastic boxes were placed in an incubator kept at 27°C in dark. On the fourth day after seed placement, Petri dishes were removed from the incubator and data on seed germination and lengths of root and shoot were collected. For each Petri dish, germination percentage was calculated by dividing total number of germinated seeds (seeds that possessed a radicle at least 1 mm in length) by total number of seeds placed and multiplied by 100. In each Petri dish, germinated seeds were separated, and for each seed, length of root and shoot were measured with a ruler to the nearest mm. The means per Petri dish were calculated.

### Statistical Analysis

To determine whether seed treatment increased plant resistance to rice water weevil, a generalized linear mixed model with Poisson distribution and log link was performed. In the model, the seed treatment was included as a fixed effect and cage as a random effect. Data on seedling emergence, plant height, number of tillers per plant, days to heading, panicle densities per plant, filled grains per panicle, percentage unfilled grains, and mass of filled grains per plant were analyzed using one-way MANOVA followed by univariate one-way ANOVA and multiple pairwise comparisons for each variable. MANOVA was used to test for overall differences among treatments because there were several dependent variables that may have been correlated, and using separate ANOVAs would have increased the risk of Type I errors. However, for one of the dependent variables (mass of filled grains), the assumption of homogeneity of variances was violated even after transformation. Although MANOVA is typically robust enough that unequal variances can be ignored, a non-parametric Welch test, which can be used in cases in which variances are unequal but distributions are normal, was also performed on filled grain mass. The dependent variable, seedling emergence, was not normal even after transformation, and hence, a Kruskal-Wallis test was conducted followed by *post-hoc* multiple comparison of means using Dunn’s test.

For the laboratory study, there were no differences in germination between controls placed in separate boxes and control dishes placed in boxes with MeJA- and JA-treated seeds, and hence, all the data from controls were pooled. Data on germination percentage, root length, and shoot length were analyzed using ANOVA followed by Tukey’s HSD multiple mean comparison test. All data were checked for normality and homogeneity in variances using Shapiro-Wilk’s test and Levene’s test, respectively. Data analyses were performed in RStudio version 1.4 (RStudio Team, 2021). All graphs were prepared in RStudio using packages “cowplot” (Wilke, 2020), “ggplot2” (Wickham, 2016), and “ggpur” (Kassambara, 2020).

## Results

### Greenhouse Experiment

**Figure 1:**
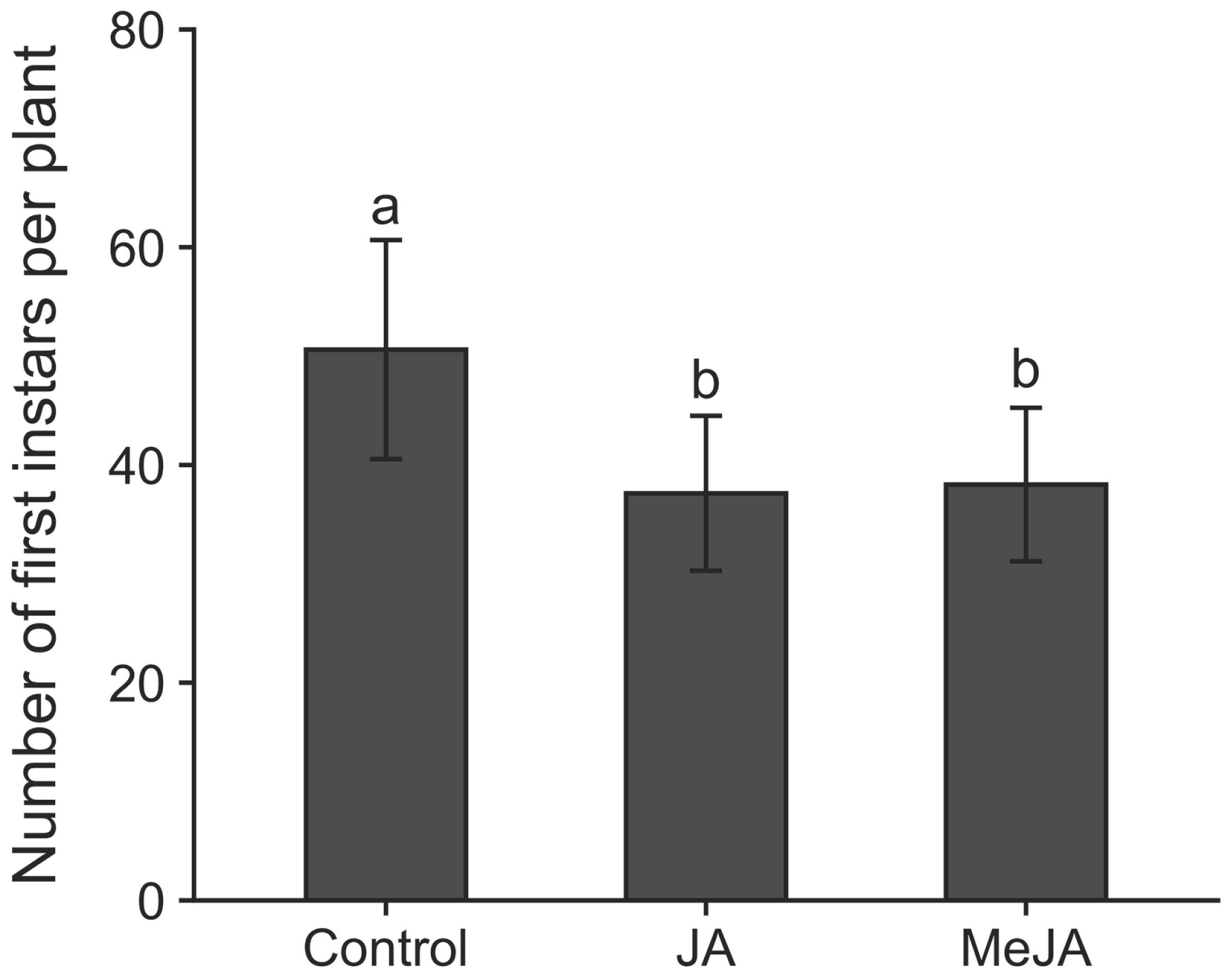
Mean (± SE) numbers of first instar rice water weevils, *Lissorhoptrus oryzophilus,* that emerged from plants grown from untreated seeds or from seeds treated with either jasmonic acid (JA; 2.5 mM) or methyl jasmonate (MeJA; 2.5 mM). Bars accompanied by different lowercase letters represent means that differed significantly (*p* < 0.05).

The numbers of first instar rice water weevils emerging from plants grown from seeds treated with MeJA (*z* = −2.94, *p* = 0.009) or JA (*z* = −3.14, *p* = 0.005) were lower than the numbers of first instars that emerged from plants grown from untreated seeds (Figure 1), verifying that seed treatment with MeJA- and JA-induced resistance to rice water weevil in rice. Seed treatment with MeJA and JA reduced emergence of rice water weevil larvae by 25 and 26%, respectively, compared to the control.

**Figure 2:**
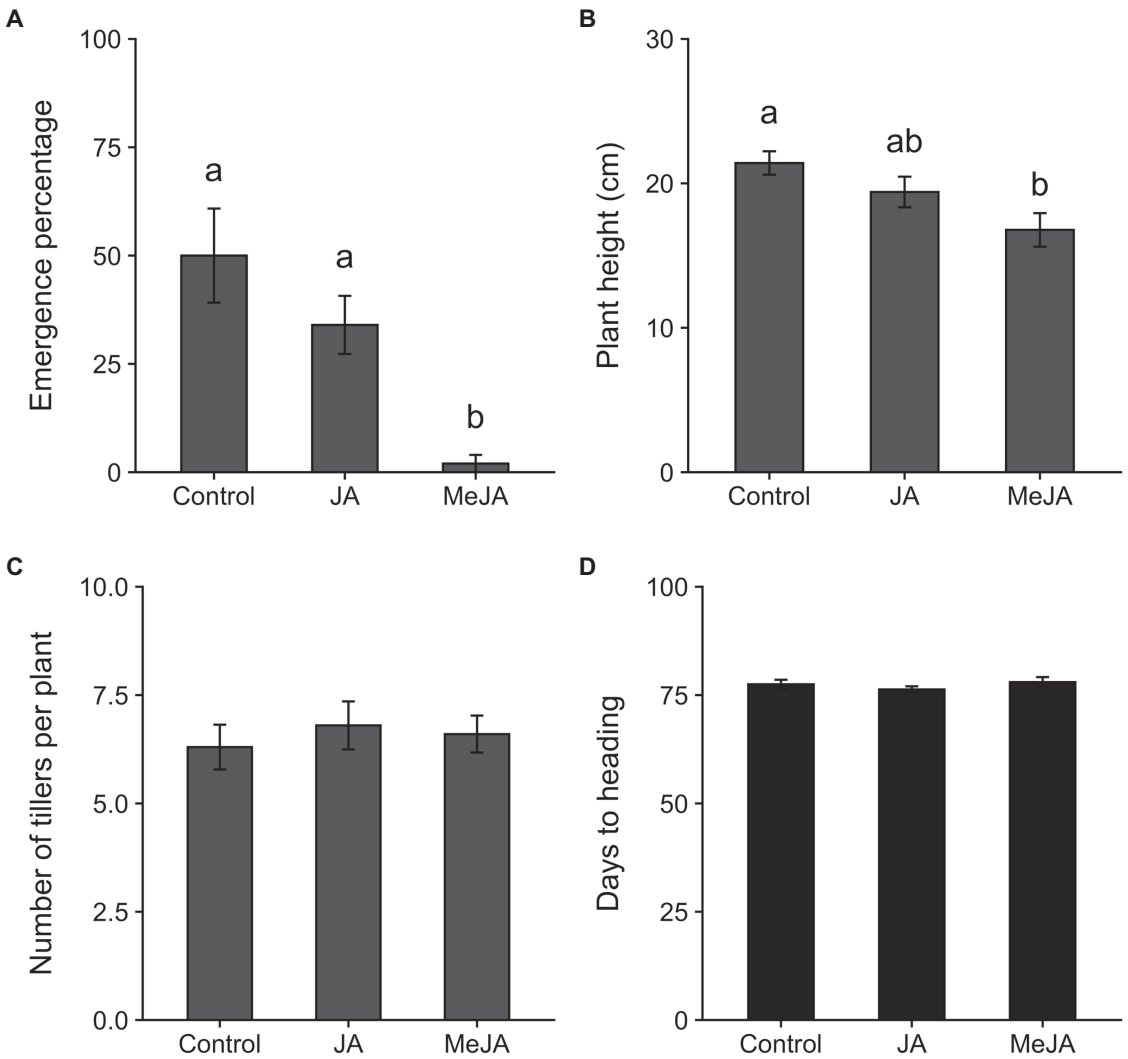
Effects of seed treatment with 2.5 mM jasmonic acid (JA), 2.5 mM methyl jasmonate (MeJA) and control (0 mM) on seedling emergence at 4 days after planting **(A)**; plant height at 14 days after planting **(B)**; numbers of tillers per plant at 45 days after planting **(C)**; and days to heading **(D)**. Bars represent means ± SE. Within each graph, different lowercase letters above the bars indicate significant difference among treatments (*p* < 0.05).

**Figure 3:**
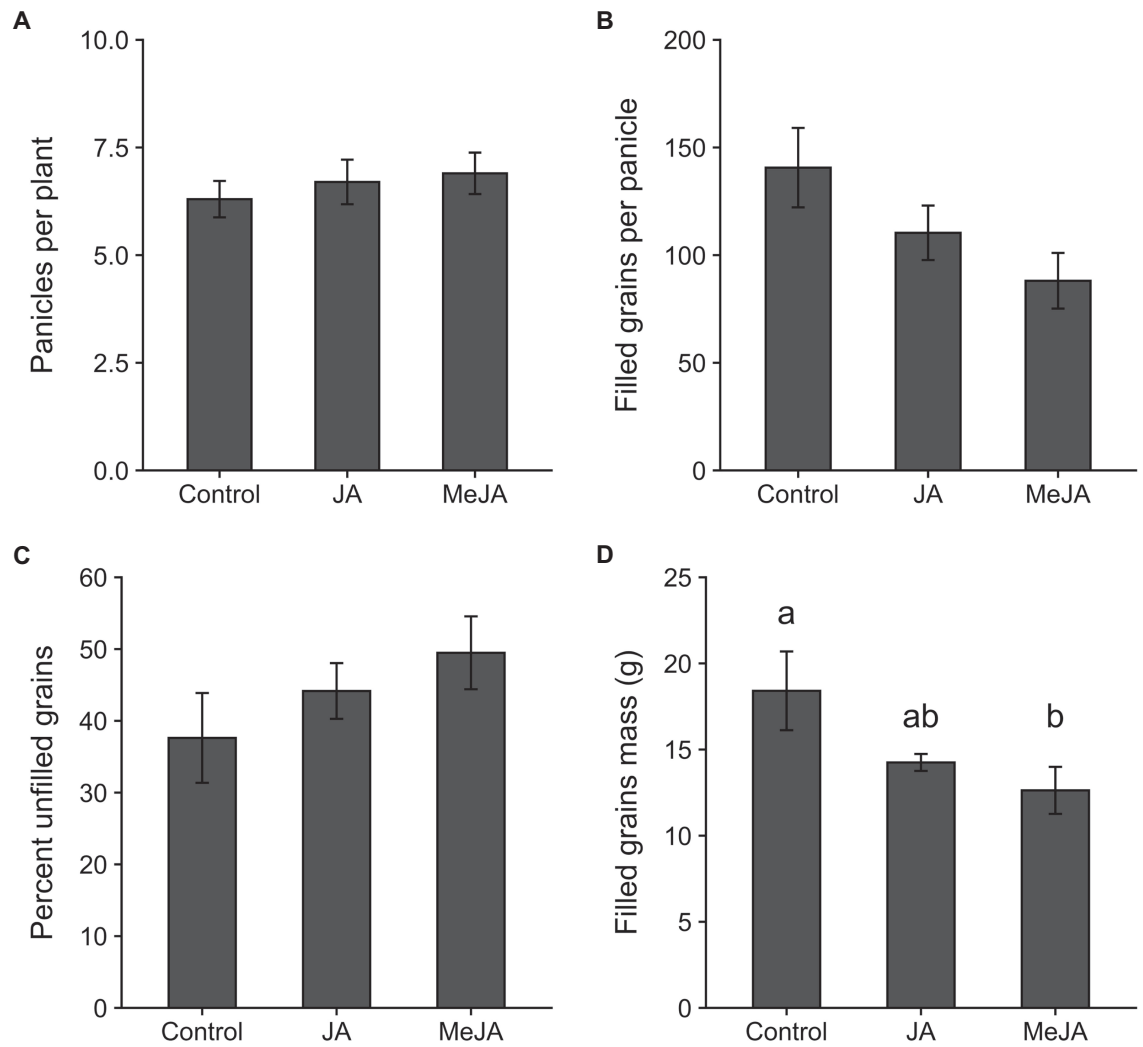
Effects of seed treatment with 2.5 mM jasmonic acid (JA), 2.5 mM methyl jasmonate (MeJA) and control (0 mM) on panicle densities per plant **(A)**; number of filled grains per panicle **(B)**; percent unfilled grains **(C)**; and filled grains mass of each plant **(D)**. Bars represent mean ± SE. Within each graph, different lowercase letters above the bars indicate significant difference among treatments (*p* < 0.05).

The results of the MANOVA revealed a significant effect of treatment on plant growth parameters (*F*_16, 42_ = 2.26, *p* = 0.02). Seed treatment with MeJA reduced the emergence of seedlings (measured at 4 days of planting) by 48% relative to control (*χ*^2^ = 14.34, *df* = 2, *p* = 0.0008; Figure 2A). The effects of seed treatment with MeJA were also detected on plant height at 14 days of planting (*F*_2, 27_ = 5.17, *p* = 0.01; Figure 2B). Plants grown from seeds treated with MeJA were approximately 22% shorter, on average, compared to those grown from control seeds. Mean numbers of tillers produced per plant at 45 days after planting were not affected by seed treatment with MeJA or JA (*F*_2, 27_ = 0.25, *p* = 0.78; Figure 2C). Plants grown from MeJA- and JA- treated seeds took similar amounts of time to reach heading as plants grown from untreated seeds (*F*_2, 27_ = 0.77, *p* = 0.47; Figure 2D). Significant effects of seed treatment with MeJA and JA were observed on one but not all yield-related traits. Total numbers of panicles on plants grown from MeJA- and JA-treated seeds did not differ significantly from numbers on control plants (*F*_2, 27_ = 0.41, *p* = 0.66; Figure 3A). There were no significant differences among treatments in numbers of filled grains per panicle (*F*_2, 27_ = 3.06, *p* = 0.06; Figure 3B) and percent unfilled grains (*F*_2, 27_ = 1.32, *p* = 0.28; Figure 3C). However, the percentages of unfilled grains were 32% higher in the MeJA treatment and 18% higher in the JA treatment relative to control. The numbers of filled grains per panicle were reduced in plants developed from seeds treated with MeJA and JA by 37 and 21%, respectively. Although the differences in numbers of filled grains per panicle and percentage of unfilled grains were not statistically significant, the combination of reductions in these two parameters resulted in a significant reduction in mass of filled grains per plant in the MeJA-treated plants compared to JA seed-treated plants and control plants (*F*_2, 27_ = 3.64, *p* = 0.04; Figure 3D). The mass of filled grains in JA-treated plants was intermediate and did not differ significantly from masses of filled grains in control- or MeJA-treated plants.

### Laboratory Study

Treatment of rice seeds with 2.5 mM JA and 2.5 mM MeJA significantly reduced percent seed germination (Table 1) at 4 days after placing seeds on germination paper. Post-germination seed development (growth of roots and shoots) was significantly reduced by seed treatment with JA and MeJA (Table 1). The reductions in lengths of root and shoot were significantly greater in the MeJA than in the JA seed treatment.

**Table 1:** Effects of seed treatment with 2.5 mM jasmonic acid (JA) and 2.5 mM methyl jasmonate (MeJA) on seed germination and post-germination seed growth in rice, *Oryza sativa* seeds.

Treatment	Germination percentage	Root length (cm)	Shoot length (cm)
Control	96.11 ± 1.11^a^	5.36 ± 0.11^a^	1.74 ± 0.05^a^
2.5 mm JA	86.67 ± 3.33^b^	2.40 ± 0.15^b^	0.87 ± 0.03^b^
2.5 mm MeJA	83.33 ± 3.33^b^	1.43 ± 0.17^c^	0.50 ± 0.00^c^
*F*	12.45	218.3	122.2
*df*	2.12	2.12	2.12
*p*	0.00	<0.0001	<0.0001

The data presented in the table are means ± SE. In each column, different lowercase letter indicates means differ significantly among treatments (*p* < 0.05).

## Discussion

The utility of elicitor seed treatment for a given crop will depend on the balance of the positive effects of elicitors (reductions in yield-reducing pest populations) and the negative effects of elicitors (reductions in yield resulting from activation of defense-related pathways). This study was undertaken to determine the possible effects of jasmonate seed treatments on the growth and yield of rice plants in an environment lacking a major pest, the rice water weevil, while also investigating whether seed treatment with MeJA and JA enhances resistance in rice against rice water weevil. As reported earlier by Kraus and Stout (2019), the application of jasmonates to rice seeds enhanced the resistance of rice plants to rice water weevils when exposed to weevils 30 days after seed treatment, when plants were producing their first tiller. Seed treatment reduced the numbers of first instar weevils emerging from plants by about 25% relative to controls, a level of induction similar to that found by Kraus and Stout (2019) in both the greenhouse and the field. Kraus and Stout (2019) also reported that MeJA-induced resistance declined over time, with resistance stronger when plants were infested at 15 days after seed treatment than at 30 days after seed treatment (as in this study). Paudel et al. (2014) reported that increased resistance by MeJA treatment in tomato plants decreased over time, but plants developed from MeJA-treated seeds were resistant to tomato fruitworm, *Helicoverpa zea* Boddie, for as long as 75 days after seed treatment.

The mechanisms of induced resistance were not investigated in this study, but treatment of seeds with JA and MeJA may have increased the activities of resistance-related enzymes or the production of secondary metabolites, such as phenolics, that in turn may have interfered with the ovipositional preference of female rice water weevils or the survival and emergence of first instar weevils from plants, or both. Elevated activities of plant defense enzymes after application of JA and MeJA have been reported in other studies. For example, Paudel et al. (2014) reported higher levels of polyphenol oxidase (PPO) in tomato plants treated as seeds with MeJA. In larch, *Larix olgensis* Henry, treatment with JA or MeJA reduced the survival and pupal weight of the gypsy moth, *Lymantria dispar* L., and these increases in resistance were associated with increased levels of plant defense proteins, including PPO, phenylalanine ammonia lyase, TI, and chymotrypsin inhibitor (Jiang and Yan, 2018). Similarly, in rice, changes in total plant phenolics and elevated activities of POD and PPO in response to feeding by the fall armyworm, *Spodoptera exigua* Hubner, have been observed (S.B., unpublished data). Further studies will be needed to determine the full range of resistance-related traits stimulated by jasmonate seed treatments in rice.

This study provided clear evidence that the induction of resistance to rice water weevils by jasmonate seed treatment was accompanied by reductions in plant growth in the absence of pests. These effects on plant growth were particularly severe immediately following treatment of seeds. In the greenhouse experiment, only 2% of seedlings had emerged from soil in the MeJA treatment at 4 days after planting, whereas 33% and 50% of seeds had emerged in the JA and control treatments, respectively. In the laboratory study, >80% of seeds had germinated in both the JA and MeJA treatments at 4 days, but the germination percentage was still lower in these treatments relative to control (Table 1). In addition, lengths of plant roots (radicles) and shoots (coleoptiles) were reduced by 50–70% by JA and MeJA treatments, with reductions in lengths greater in the MeJA treatment than in the JA treatment. Taken together, the results of both the laboratory and greenhouse experiments indicate that JA and MeJA at the tested concentration inhibit or delay germination but do not ultimately reduce seed germination. Similarly, delays in seed germination and seedling emergence and decreased root and shoot growth in various agricultural crops due to application of MeJA or JA have been reported in several studies (Norastehnia et al., 2007; Paudel et al., 2014).

Although the effects of jasmonate seed treatment on the germination and early growth of rice plants were quite severe, the effects on growth appeared to erode over time. At 14 days after planting, plants in the MeJA treatment were significantly shorter than controls, but the same was not true of plants in the JA treatment. Reductions in plant heights may have been caused by delays in seedling emergence rather the costs of MeJA application *per se*. At 45 days after planting, the numbers of tillers per plant were unaffected by seed treatment with JA or MeJA. Similarly, days until exertion of the first panicle (heading) did not differ among treatments. Diminished effects of exogenous JA and MeJA over time have been observed in a few other crops. In corn, Feng et al. (2012) reported that JA application to above- and below-ground portions of plants resulted in reductions in root length, root surface area, and root biomass but increased the levels of the plant defense compounds, DIMBOA, and phenolics, 2 weeks after application. However, the effects on plant growth were not detected 4 weeks after application. Moreover, 4 weeks after treatment, shoot and root biomasses of JA-treated plants were greater relative to untreated plants indicating no apparent defense allocation costs. Similarly, treatment of tomato seeds with JA reduced plant heights but had no long-term effects on plant growth (Worrall et al., 2012). Our results indicate that JA and MeJA seed treatment in rice had a strong inhibitory effect on plant growth immediately after treatment but that the effects of JA and MeJA diminish over time, such that growth in 6-week-old plants had fully recovered.

The most critical potential impacts of jasmonate treatments on plants are those involving negative effects on crop yield. In the current study, four components of yield – panicles per plant, total number of filled grains per panicle, percent unfilled grains, and mass of filled grains per plant – were measured. The first three yield components were not significantly affected by seed treatment with jasmonates, although the numbers of filled grains per panicle trended lower in jasmonate treatments, while percent unfilled grains trended higher in treated plants. In contrast, masses of filled grains per plant, which is a composite of the other three yield components, were reduced (*p* = 0.04) in the MeJA treatment relative to JA and control treatments when MANOVA followed by pairwise comparisons was performed. The results of the non-parametric Welch test showed no differences in masses of filled grains among JA, MeJA, and control treatments. Thus, it appears that decreases in numbers of filled grains per panicle, increases in percent unfilled grains, and perhaps other effects (e.g., average grain weights) combined to result in marginal decreases in overall yields in plants treated with MeJA as seeds, with yields intermediate (but not significantly different from the control treatment) in JA-treated plants.

Constitutive expression of jasmonate-induced defenses is costly to plants. For example, Cipollini (2007) showed that overexpression of *At*JMT (jasmonic acid carboxyl methyltransferase – an enzyme that methylates JA to MeJA) in *Arabidopsis thaliana* (L.) Heynh resulted in development of stunted plants and reduced seed germination and yields measured as total seed mass. Likewise, Kim et al. (2009), in rice, showed that overexpression of JMT, which increased the levels of MeJA in young panicles, had a drastic effect on rice yield due to decreased numbers of spikelet per panicle and lower filling rates that ultimately resulted in lower grain masses. The above studies attributed decreases in plant yield/fitness to the overproduction of MeJA. In contrast to constitutive resistance, induced resistance, in which resistance-related traits are expressed only when plants are subjected to biotic and abiotic stresses, allows plants to use their limited resources for growth and reproduction when defenses against stresses are not needed. Dietrich et al. (2005) reported that given the time and resources, *A. thaliana* plants treated with the chemical elicitor BION [benzo (1,2,3) thiadiazole-carbothioic acid S-methylester, a mimic of salicylic acid and elicitor of induced resistance against biotrophic pathogens] were able to recover from a single application of chemical elicitor without losses in yield.

Other studies that have investigated the effects of application of MeJA and JA on plant yields have found contrasting results. In tomato, repeated applications of JA at 15 day intervals until harvest resulted in a delay in time to fruit set and reduced the number of fruits per plant, fruit weight, and number of seeds per plant (Redman et al., 2001). Likewise, Agrawal et al. (1999) reported that JA-induced responses reduced the plant fitness in the absence of herbivory in terms of time to first flower formation and number of pollen per flower but not in seed number and seed weight. However, Thaler (1999) reported that, regardless of the presence of herbivory, foliar sprays of JA reduced the number of flowers produced in treated plants relative to untreated plants but had no effect on fruit weight and number of fruits produced per plant. In tomato, seed treatment with 3 mM JA did not reduce the fruit dry weights (Worrall et al., 2012). These differences in JA/MeJA effects of plant fitness and yield are probably due to the differences in concentrations of elicitors, application methods (foliar and seed treatment), time and frequency of elicitor application, plant stage, days to senescence, and levels of herbivory.

The inhibitory effects on seed germination, plant height, and yields were stronger in MeJA-treated plants compared to the JA-treated plants. One possible reason for these differences is that MeJA more effectively reached its target sites in the seed. This may have been the case because the different polarities of the two compounds allowed MeJA to more easily penetrate into rice seeds. Alternatively, because MeJA is a volatile compound and has a lower vapor pressure than JA, it may have penetrated faster and moved more quickly through plant tissues (Farmer and Ryan, 1990; Seo et al., 2001) and more quickly elicited plant defense genes compared to JA (Jiang and Yan, 2018). However, it is important to note that these different effects on growth and yield occurred despite the fact that JA- and MeJA-induced resistance to rice water weevil to roughly equivalent extents, which suggests that different jasmonate elicitors may have differing effects on plant growth, yield, and defense. Qi et al. (2016) found that mutant *os*JMT1 rice plants that overexpress the jasmonic acid carboxyl methyltransferase gene had high levels of MeJA and low levels of JA and JA-Ile. The *os*JMT1 rice lines were resistant to brown planthopper, *Nilaparvatha lugens* Stal nymphs, but adult females were more attracted to *os*JMT1 rice lines compared to wild types, perhaps because of low levels of JA. However, in *os*JMT1 rice lines, plant height and yields were reduced relative to the wild type. The authors suggested that MeJA might have a greater role in plant growth and development than in plant defense, while JA and JA-Ile might be more involved in defense against pathogens and insects.

Explanations for reduced growth and yields in jasmonate-treated plants generally fall into two categories. On the one hand, the applications of jasmonates can result in increased allocation of resources to resistance-related pathways, processes, and metabolites at the expense of growth and reproduction (Walters and Heil, 2007; Guo et al., 2018). On the other hand, negative effects of JA and MeJA treatment on growth and development could result from hormonal cross talk between hormones and hormonal pathways, such as those involving JA and gibberellic acid (GA). Upregulation of the JA signaling pathway has been shown to downregulate the GA pathway (Hou et al., 2013). Downregulation of GA, for example, might have impacted rice germination and seedling growth in two ways in the current study. First, GA regulates the growth rate of plant tissues by affecting cell proliferation and expansion (Achard et al., 2009; Ubeda-Tomás et al., 2009). Second, in cereal crops, upon imbibition of water, GA present in the seeds induces the expression of an α-amylase gene and other hydrolytic enzymes and proteases involved in germination (Sugimoto et al., 1998). During germination, α-amylase plays a critical role in degradation of the insoluble starch granules to soluble sugars and mobilization of energy reserves to the plant tissues (Palmiano and Juliano, 1972), which are then utilized for growth and elongation of embryonic roots and shoots (Nomura et al., 1969; Jones and Jacobsen, 1991). Treatment of seeds with MeJA decreased activity of α-amylase in germinating seeds that led to a delay in seed germination and reduction in seedling root elongation in corn, *Zea mays* L. (Norastehnia et al., 2007). Similarly, Lahuta et al. (2018) reported that in triticale (*Triticosecale* Witmmack), reduced seed germination, lower fresh and dry weight of embryo and fewer root hairs on embryonic roots were associated with decreased starch degradation by α-amylase in the presence of MeJA.

The results of this current study help clarify results from the recently published field study (Kraus and Stout, 2019) on induction of resistance to rice water weevils by MeJA seed treatment. In this prior study, treatment of rice seeds by MeJA resulted in an approximately 30% reduction in densities of rice water weevil larvae on the roots of rice plants, but reductions in weevil densities by MeJA treatment were not as large as those obtained by treating plants multiple times with a pyrethroid. Reductions or delays in seedling emergence and plant growth resulting from MeJA seed treatment were similar to those observed in the current study. Yields (panicle masses) did not differ between untreated and MeJA-treated plants, but yields were higher from insecticide-treated plots than from MeJA-treated plots. However, it was unclear whether the lower yields from MeJA-treated plots were the result of higher densities of yield-reducing weevil larvae in MeJA-treated plots relative to insecticide-treated plots, or from direct effects of MeJA on plant yield. The results of the current study, which demonstrates reductions in yield in MeJA-treated plants in the absence of weevils, suggest that the lower yields in MeJA-treated plots than in insecticide-treated plots in Kraus and Stout (2019) may have resulted partly from the direct effects of MeJA on plant yield. Further work will be needed to define the conditions under which the benefits of reduced rice water weevil densities in MeJA-treated plants outweigh the costs associated with the treatment. However, the marginal reductions in yield, including the non-significant effect of JA on yield, and the temporary nature of reductions in growth caused by jasmonate treatment in this study suggest that use of elicitors could be integrated into management programs for pests in rice under certain circumstances.

Plant elicitors applied to seeds may be advantageous as they are simple to implement and are not accompanied by the same deleterious effects on non-target organisms and the environment as is the use of insecticides. Further research on the positive effects of elicitor seed treatments on plant defense under field conditions is warranted, including possible activation of indirect defenses and defense priming mechanisms by elicitors. However, cost-effective methods for applying elicitors to seeds will need to be developed. In addition, cultural practices (e.g., seeding rates) may need to be adjusted to compensate for the negative effects of elicitors on plant growth. Finally, as noted above, further research on different crops under field conditions will be needed to further define those situations in which the benefits accruing from reduced densities of pests outweigh reductions in yield resulting from activation of plant defenses, which are likely to be species-specific.

## Data Availability Statement

The original contributions presented in the study are included in the article/supplementary material, and further inquiries can be directed to the corresponding author.

## Author Contributions

MS conceived the study. SB and MS designed the study and reviewed and edited the manuscript. SB conducted the study, collected and analyzed the data, and wrote the first draft of the manuscript. All authors contributed to the article and approved the submitted version.

## Conflict of Interest

The authors declare that the research was conducted in the absence of any commercial or financial relationships that could be construed as a potential conflict of interest.

## Publisher’s Note

All claims expressed in this article are solely those of the authors and do not necessarily represent those of their affiliated organizations, or those of the publisher, the editors and the reviewers. Any product that may be evaluated in this article, or claim that may be made by its manufacturer, is not guaranteed or endorsed by the publisher.
